# Potential and Limitations of an Improved Method to Produce Dynamometric Wheels

**DOI:** 10.3390/s18020541

**Published:** 2018-02-10

**Authors:** José Luis Bueno-López, Jesús Cardenal, Álvaro Deibe, Javier García de Jalón

**Affiliations:** 1 INSIA, Universidad Politécnica de Madrid, Carretera de Valencia km. 7, 28031 Madrid, Spain; 2 Vehicle Dynamics Group, University of A Coruña, 15403 Ferrol, Spain; jcarde@udc.es; 3 Integrated Group for Engineering Research, University of A Coruña, 15403 Ferrol, Spain; adeibe@udc.es; 4 ETSII and INSIA, Universidad Politécnica de Madrid, José Gutiérrez Abascal 2, 28006 Madrid, Spain; javier.garciadejalon@upm.es

**Keywords:** measuring wheel, dynamometric wheel, wheel transducer, forces measurement, tyre/road contact

## Abstract

A new methodology for the estimation of tyre-contact forces is presented. The new procedure is an evolution of a previous method based on harmonic elimination techniques developed with the aim of producing low cost dynamometric wheels. While the original method required stress measurement in many rim radial lines and the fulfillment of some rigid conditions of symmetry, the new methodology described in this article significantly reduces the number of required measurement points and greatly relaxes symmetry constraints. This can be done without compromising the estimation error level. The reduction of the number of measuring radial lines increases the ripple of demodulated signals due to non-eliminated higher order harmonics. Therefore, it is necessary to adapt the calibration procedure to this new scenario. A new calibration procedure that takes into account angular position of the wheel is completely described. This new methodology is tested on a standard commercial five-spoke car wheel. Obtained results are qualitatively compared to those derived from the application of former methodology leading to the conclusion that the new method is both simpler and more robust due to the reduction in the number of measuring points, while contact forces’ estimation error remains at an acceptable level.

## 1. Introduction

Tyres are an important element in road vehicles because of the influence they have on longitudinal and lateral dynamics, damping of vibrations induced by road surface irregularities and in transmitting the normal loads to the ground. Tyre-road contact forces and moments play an important role when studying the dynamic behaviour of the vehicle and attempting to improve vehicle handling, riding comfort and even vehicle safety.

Knowledge of the contact forces and moments could be used to validate the vehicle’s mathematical models, checking their accuracy with respect to reality [1]. Another field where this knowledge would be potentially advantageous is in improving current active safety systems such as ABS (Anti-lock Brake System), ESP (Electronic Stability Program) and TCS (Traction Control System). These systems rely on the indirect estimation of the contact forces and moments and other dynamic variables to detect, prevent and minimize dangerous situations that can arise while driving the vehicle [2,3,4,5,6,7,8]. Consequently, the direct measurement of the aforementioned tyre-road contact forces would allow a more direct and reliable approach to be taken, as well as reducing the degree of complexity of the electronic control units, since it would be no longer necessary to use complicated estimation algorithms [9].

However, even though there are commercial dynamometric wheels (or wheel force transducers) available for the direct measurement of the contact forces, their cost is sometimes higher than that of the vehicle itself. This excessive cost effectively prevents their use in production vehicles, limiting the possibility for further improvement of active vehicle safety systems [10,11]. These commercial dynamometric wheels are modular for the sake of versatility and can be used with a wide range of hub configurations. They are composed of an adaptor part (*modified rim* or *hub adaptor*) where the six-axis wheel force transducer is mounted. Once assembled, it can be handled as a normal wheel.

However, this approach has a disadvantage that when the system where measurements are being taken is modified in a non-negligible way, the wheel’s moments of inertia and the unsprung mass will not be the same as those of the real system. Therefore, the contact forces measured by these commercial systems can differ significantly from real working conditions.

In [12], a new method was proposed which worked around the disadvantages of most of the existing commercial dynamometric wheels by instrumenting the standard wheels of vehicles. In this case, although the unsprung mass is slightly increased, the dynamic behaviour is not significantly altered. The method presented a linear combination of the strain signals obtained in different radial lines of the same measurement circumference, with the aim of obtaining a ‘continuous’ signal independent of the wheel rotation angle and proportional to the force applied. For the application of the method, it is necessary that higher order non-eliminated harmonics are negligible compared to the dominant one. The method was applied to a ten-spoke wheel and the previous condition was met. However, it was also suggested that fewer measuring radial lines would reduce the number of higher harmonics eliminated. Given that commercial rims do not usually have a high number of spokes, the interest of studying a wheel with fewer radial lines became apparent.

A scenario with a reduced number of radial lines was studied in [13] using the same ten-spoke wheel but only considering strain signals from five of the ten radial lines. In addition, improvements to the aforementioned method by relaxing the symmetry conditions needed in order to cancel out more harmonics were presented. Even in [12], the strain signals measured were not completely equivalent, mainly due to the difficulty of sticking the gauges in precise and exact locations. Not fulfilling the symmetry requirements produces a higher ripple on the ‘continuous’ signals. This would also result in an increase of the estimation error because the higher order harmonics would be less and less negligible. In order to solve this problem and to reduce the estimation error of contact forces, an improvement to the method consisting of using a calibration matrix dependent on the angle rotated by the wheel instead of using one with constant values was proposed in [13].

Another improvement presented in [13] is the possibility to reduce the number of circumferences from three to two without a significant change in the estimation error. This was possible by giving more importance to the signals where the second order harmonic is dominant. These improvements have been patented (Patent ES2566048).

In this paper, these improvements are explained in a simplified manner and applied to a five-spoke commercial wheel.

## 2. Description of the Measurement System

### 2.1. Measurement Method

**Figure 1:**
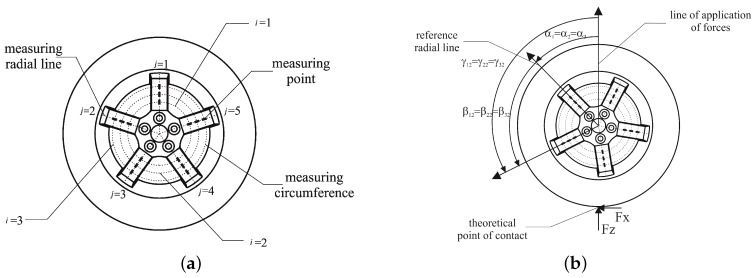
Definition of the notation used. (**a**) definition of measuring circumference and measuring radial line; (**b**) definition of angular coordinates.

The relationship between the strain signal $\varepsilon_{ij}$ obtained at the measuring circumference *i* and at the measuring radial line *j* (see Figure 1a) and the forces and moments is assumed to be linear. The linear coefficients, or influence functions, are periodic over the angle rotated by the wheel and thus can be expressed as a Fourier series expansion. This equation already presented in [12,13] has been generalized assuming that the symmetry and antisymmetry conditions of the influence functions are no longer enforced, relaxing consequently the symmetry requirements of the rim and taking into account the minimal displacements while sticking the gauges to the rim.

(1) $$\begin{array}{cc} \varepsilon_{ij}\left(\gamma_{ij},t\right) & =F_{X}\left(t\right)\varphi_{ij}^{X}\left(\gamma_{ij}\right)+F_{Y}\left(t\right)\varphi_{ij}^{Y}\left(\gamma_{ij}\right)+F_{Z}\left(t\right)\varphi_{ij}^{Z}\left(\gamma_{ij}\right)+\\ & +M_{X}\left(t\right)\psi_{ij}^{X}\left(\gamma_{ij}\right)+M_{Y}\left(t\right)\psi_{ij}^{Y}\left(\gamma_{ij}\right)+M_{Z}\left(t\right)\psi_{ij}^{Z}\left(\gamma_{ij}\right)+\\ & +\zeta_{i}\left(t\right)=\\ & =F_{X}\left(t\right)\sum_{k=0}^{\infty }\left(A_{ijk}^{f_{4}}\cos\left(k\gamma_{ij}\right)+B_{ijk}^{f_{4}}\sin\left(k\gamma_{ij}\right)\right)+\\ & +F_{Y}\left(t\right)\sum_{k=0}^{\infty }\left(A_{ijk}^{f_{2}}\cos\left(k\gamma_{ij}\right)+B_{ijk}^{f_{2}}\sin\left(k\gamma_{ij}\right)\right)+\\ & +F_{Z}\left(t\right)\sum_{k=0}^{\infty }\left(A_{ijk}^{f_{3}}\cos\left(k\gamma_{ij}\right)+B_{ijk}^{f_{3}}\sin\left(k\gamma_{ij}\right)\right)+\\ & +M_{X}\left(t\right)\sum_{k=0}^{\infty }\left(A_{ijk}^{f_{1}}\cos\left(k\gamma_{ij}\right)+B_{ijk}^{f_{1}}\sin\left(k\gamma_{ij}\right)\right)+\\ & +M_{Y}\left(t\right)\sum_{k=0}^{\infty }\left(A_{ijk}^{f_{5}}\cos\left(k\gamma_{ij}\right)+B_{ijk}^{f_{5}}\sin\left(k\gamma_{ij}\right)\right)+\\ & +M_{Z}\left(t\right)\sum_{k=0}^{\infty }\left(A_{ijk}^{f_{6}}\cos\left(k\gamma_{ij}\right)+B_{ijk}^{f_{6}}\sin\left(k\gamma_{ij}\right)\right)+\\ & +\zeta_{i}\left(t\right) \end{array}$$

(2) $$\gamma_{ij}=\alpha_{i}+\beta_{j},$$

where

- $F_{X}$, $F_{Y}$ and $F_{Z}$ are, respectively, the *X*, *Y* and *Z* components of the contact force.
- $M_{X}$, $M_{Y}$ and $M_{Z}$ are, respectively, the *X*, *Y* and *Z* components of the contact moment.
- $\varepsilon_{ij}$ is the strain measured at the circumference *i* and the measuring radial line *j* in the radial direction.
- $\varphi_{ij}^{X}$, $\varphi_{ij}^{Y}$ and $\varphi_{ij}^{Z}$ are the *influence functions* that account for the effect of the forces $F_{X}$, $F_{Y}$ and $F_{Z}$ on the strain $\varepsilon_{ij}$. The influence functions represent the physical concept of the strain generated by a unit force and therefore are an indirect measure of the stiffness of the rim. Its units are με/N or also μm/(m·N).
- $\psi_{ij}^{X}$, $\psi_{ij}^{Y}$ and $\psi_{ij}^{Z}$ are the *influence functions* that show the effect of the moments $M_{X}$, $M_{Y}$ and $M_{Z}$ on the strain $\varepsilon_{ij}$. These influence functions are usually determined by means of a calibration process: a set of static tests applying known forces and moments and registering the strains obtained at the points of measurement. They may also be obtained by means of a finite element analysis if a good enough model is available for the rim being studied.
- $A_{ijk}^{f_{1}}$ is the amplitude of the *k* cosine term of the strain signal generated in the measuring radial line *j* and the measuring circumference *i* by a $F_{X}$ unit force applied on the contact patch. Similarly, $A_{ijk}^{f_{2}}$, $A_{ijk}^{f_{3}}$, $A_{ijk}^{f_{4}}$, $A_{ijk}^{f_{5}}$ and $A_{ijk}^{f_{6}}$ are the amplitudes of the *k* cosine term of the strain signals generated at the measuring point ij by $F_{Y}$, $F_{Z}$, $M_{X}$, $M_{Y}$ and $M_{Z}$ unit stresses, respectively.
- $B_{ijk}^{f_{1}}$ is the amplitude of the *k* sine term of the strain signal generated in the measuring radial line *j* and the measuring circumference *i* by a $F_{X}$ unit force applied on the contact area. Similarly, $B_{ijk}^{f_{2}}$, $B_{ijk}^{f_{3}}$, $B_{ijk}^{f_{4}}$, $B_{ijk}^{f_{5}}$ and $B_{ijk}^{f_{6}}$ are the amplitudes of the *k* sine term of the strain signals generated at the measuring point ij by $F_{Y}$, $F_{Z}$, $M_{X}$, $M_{Y}$ and $M_{Z}$ unit stresses, respectively.
- $\gamma_{ij}$ is the angle between the line where the forces are applied and the radial line *j*.
- $\alpha_{i}$ is the angle between the line where the forces are applied and the reference radial line.
- $\beta_{j}$ is the angle between the radial line *j* and the reference radial line (see Figure 1b for details).
- $\zeta_{i}$ comprehends all the factors that do not depend on the angular position $\gamma_{ij}$ such as temperature, centrifugal forces and pressure.

If the measuring point *j* of the measuring circumference *i* is placed exactly on a plane of symmetry of the wheel, the terms $A_{ijk}^{f_{1}}$, $A_{ijk}^{f_{5}}$, $A_{ijk}^{f_{6}}$, $B_{ijk}^{f_{2}}$, $B_{ijk}^{f_{3}}$ and $B_{ijk}^{f_{4}}$ would be null. This is consistent with [12], where there are certain forces and moments ($F_{X}$, $M_{Y}$ and $M_{Z}$) that generate antisymmetric strain signals and, as a result, only sine terms appear in the Fourier series expansion. Inversely, the forces and moments $M_{X}$, $F_{Y}$ and $F_{Z}$ generate symmetric strain signals and only cosine terms appear in the Fourier series expansion. Only in the case where all the strain gauges of the same measuring circumference are bonded at the same radial distance can the same signals then be obtained but with a phase difference, so that $A_{i1k}^{f_{m}}=A_{i2k}^{f_{m}}=\cdots =A_{in_{r}k}^{f_{m}}$ and $B_{i1k}^{f_{m}}=B_{i2k}^{f_{m}}=\cdots =B_{in_{r}k}^{f_{m}}$ for m=1,2,⋯,6. Consequently, all the strain signals of the same measuring circumference would have the same harmonic composition.

For the application of the measurement method, the strain signals of the same circumference *i* are combined to annul the effect of $\gamma_{ij}$. In [12], it is shown that the first harmonic is generally the dominant one in the strain signals generated by the different forces and moments, except for the moment acting on the *y*-axis, which generates strain signals in which the first harmonic is approximately null and the second harmonic is the dominant one. Therefore, signals proportional to the amplitude of the first and second harmonic of the strains are obtained.

It is proved in [13] that the optimal combinations of the strain signals obtained at the same measuring circumference coincide with the ones presented in [12], with the difference being that these signals will not be constant with respect to the rotation angle and a non-negligible ripple will appear. The following combinations must be made to compute the signals where the first harmonic is the dominant one, $E_{Si}$ and $E_{Ai}$:

(3) $$E_{Si}\left(t\right)=\frac{2}{n_{r}}\sum_{j=1}^{n_{r}}\left(\varepsilon_{ij}\left(\gamma_{ij},t\right)\cdot \cos\left(\alpha_{i}+\left(j-1\right)\frac{2\pi }{n_{r}}\right)\right),$$

(4) $$E_{Ai}\left(t\right)=\frac{2}{n_{r}}\sum_{j=1}^{n_{r}}\left(\varepsilon_{ij}\left(\gamma_{ij},t\right)\cdot \sin\left(\alpha_{i}+\left(j-1\right)\frac{2\pi }{n_{r}}\right)\right).$$

$n_{r}$ denotes the number of measuring radial lines. $n_{S}$ and $n_{A}$ denote the number of circumferences where signals $E_{Si}$ and $E_{Ai}$ are computed. $n_{S}^{\prime }$ and $n_{A}^{\prime }$ are the number of circumferences where the signals whose dominant harmonic is the second one, $E_{Si}^{{}^{\prime }}$ and $E_{Ai}^{{}^{\prime }}$, are calculated. The latter can be obtained as follows:

(5) $$E_{Si}^{{}^{\prime }}\left(t\right)=\frac{2}{n_{r}}\sum_{j=1}^{n_{r}}\left(\varepsilon_{ij}\left(\gamma_{ij},t\right)\cdot \cos\left(2\left(\alpha_{i}+\left(j-1\right)\frac{2\pi }{n_{r}}\right)\right)\right),$$

(6) $$E_{Ai}^{{}^{\prime }}\left(t\right)=\frac{2}{n_{r}}\sum_{j=1}^{n_{r}}\left(\varepsilon_{ij}\left(\gamma_{ij},t\right)\cdot \sin\left(2\left(\alpha_{i}+\left(j-1\right)\frac{2\pi }{n_{r}}\right)\right)\right).$$

### 2.2. Dependence of the Demodulated Signals *E* with the Wheel Rotation

**Figure 2:**
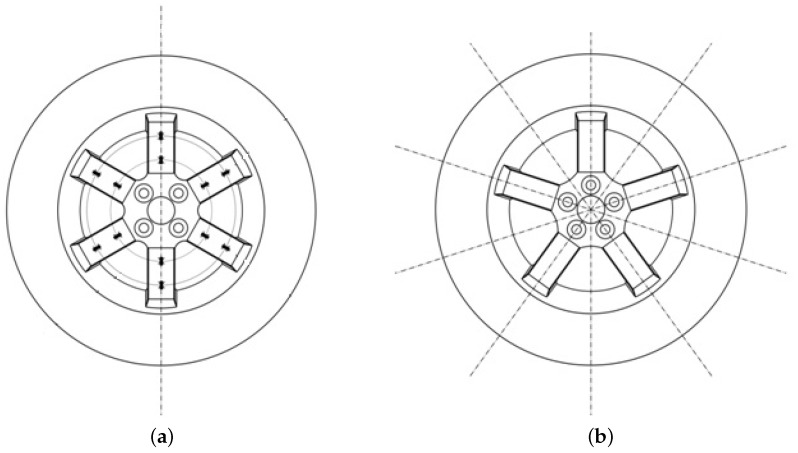
Planes of symmetry on the rims. (**a**) case where the rim has only one plane of symmetry; (**b**) case where the rim has five planes of symmetry.

The previous signals, $E_{S}$, $E_{A}$, $E_{Si}^{{}^{\prime }}$ and $E_{Ai}^{{}^{\prime }}$, are theoretically continuous and therefore independent of the rotation angle of the wheel. However, as explained in [12], these signals present some other harmonics besides the first (for signals $E_{Si}$ and $E_{Ai}$) and the second one (for signals $E_{Si}^{{}^{\prime }}$ and $E_{Si}^{{}^{\prime }}$). This effect is increased when the measuring points are not located exactly on planes of symmetry, either because these simply do not exist due to the bolt pattern, as shown in Figure 2, or because the signals measured at different radial lines are not equivalent due to minor deviations introduced during the rim manufacturing process or to the small random errors made when bonding the strain gauges. Since this task is usually done manually, it is very difficult to place the sensors exactly at the required position. Nonetheless, if the ratio between the non-eliminated higher order harmonics and the dominant one from which the forces and moments will be computed is negligible, then these signals could be assumed to be constant and the dependence on the rotation angle of the wheel would be cancelled. This is the case of the wheel presented in the previous paper [12].

However, in some cases, such a ratio cannot be considered negligible and, consequently, if these signals were assumed to be independent of the angle of rotation, the error made in the estimation of contact forces and moments would be large. The probability of such being the case will be higher when using a lower number of measuring radial lines because, as it is shown in [13], the fewer radial lines used for measurement, the fewer higher order harmonics that can be eliminated.

Therefore, an improvement in [13] was proposed: instead of using a constant calibration matrix, each element of which is obtained as the ratio between the average value of the corresponding signal *E* and the force applied, as is explained in [12], the improved method uses a variable calibration matrix dependent on the angular position of the wheel.

### 2.3. Computation of the Calibration Matrices

The method of computation of the calibration matrices is very similar to that explained in [12]. A set of static tests is carried out, where the forces applied are known and the strains in the measuring points are recorded for various different equidistant angular positions for a complete rotation of the wheel. Employing (1) with sufficient data, the influence functions, $\varphi_{ij}^{X}$, $\varphi_{ij}^{Y}$, $\varphi_{ij}^{Z}$, $\psi_{ij}^{X}$, $\psi_{ij}^{Y}$ and $\psi_{ij}^{Z}$, can be obtained (usually, more data is available than the minimum necessary to solve the system of equations and therefore the system is solved by minimizing the square-root error). These influence functions describe the strain field of the rim when the corresponding unit force is applied and they depend on the angle rotated by the wheel.

The problem to be solved is finding the linear application, which relates the demodulated signals *E* with the forces actuating on the wheel:

(7) $$f\left(t\right)=B\left(\alpha \right)e\left(\alpha ,t\right),$$

where $f\left(t\right)$ is the vector containing the contact forces and moments. Due to the distinction between symmetric and antisymmetric stress introduced in [12], the order of the elements is the following:

(8) $$f\left(t\right)={\left[\begin{array}{cccccc} M_{X}\left(t\right) & F_{Y}\left(t\right) & F_{Z}\left(t\right) & F_{X}\left(t\right) & M_{Y}\left(t\right) & M_{Z}\left(t\right) \end{array}\right]}^{T},$$

and

(9) $$\begin{array}{cc} e\left(\alpha ,t\right)= & [E_{S1}\left(\alpha ,t\right)\;E_{A1}\left(\alpha ,t\right)\;\cdots \;E_{Sn_{1}}\left(\alpha ,t\right)\;E_{An_{1}}\left(\alpha ,t\right)\\ & E_{S1}^{{}^{\prime }}\left(\alpha ,t\right)\;E_{A1}^{{}^{\prime }}\left(\alpha ,t\right)\;\cdots \;E_{Sn_{2}}^{{}^{\prime }}\left(\alpha ,t\right)\;E_{An_{2}}^{{}^{\prime }}\left(\alpha ,t\right){]}^{T}, \end{array}$$

where $n_{1}$ is the number of measuring circumferences where the first harmonic dependent signals $E_{Si}$ and $E_{Ai}$ are computed and $n_{2}$, the number of measuring circumferences where the second harmonic dependent signals, $E_{Si}^{{}^{\prime }}$ and $E_{Ai}^{{}^{\prime }}$ are computed. $n_{e}=2n_{1}+2n_{2}$ will denote the number of elements in vector e.

Bearing in mind that the influence functions previously obtained are in fact the unit strain per unit force applied, it is possible to calculate the demodulated signals associated with each of these strains. For example, if the influence function $\varphi_{ij}^{Z}$ corresponding to the $F_{Z}$ force were considered, the corresponding $f\left(t\right)$ vector would be:

(10) $$f\left(t\right)={\left[\begin{array}{cccccc} 0 & 0 & 1 & 0 & 0 & 0 \end{array}\right]}^{T}.$$

Hence, all the influence functions can be collected in the following matrix equation:

(11) $$B{\left(\alpha \right)}_{6\times n_{e}}A\left(\alpha \right)=I_{6},$$

where $A\left(\alpha \right)$ represents the matrix composed of the vectors $e\left(\alpha \right)$ of each influence function arranged by columns and I is the 6×6 identity matrix. It should be noted that the influence functions are not time dependent. Time dependence is a consequence of the variation of the force, $f\left(t\right)$, but since the influence functions represent the strain per unit force, dependence on time no longer applies.

Finally, the calibration matrix $B\left(\alpha \right)$ is obtained as the Moore–Penrose pseudoinverse of $A\left(\alpha \right)$.

The procedure explained here is the same as that used in [12]. Previously, matrix A in Equation (11) was composed of the average values of $e\left(\alpha \right)$ over α because the signals were considered constant and independent of the rotation of the wheel, considering the ripple negligible. In [13], it was proposed solving (11) for every angular position α considered in the static calibration tests. For angular positions in-between, the elements of the intermediate matrix can be obtained through an interpolation process based on Newton’s divided differences interpolation polynomial. This step introduces little computational complexity because the angular position is already known, since it was also needed to compute the signals e.

### 2.4. Relationship between the Influence Functions

**Figure 3:**
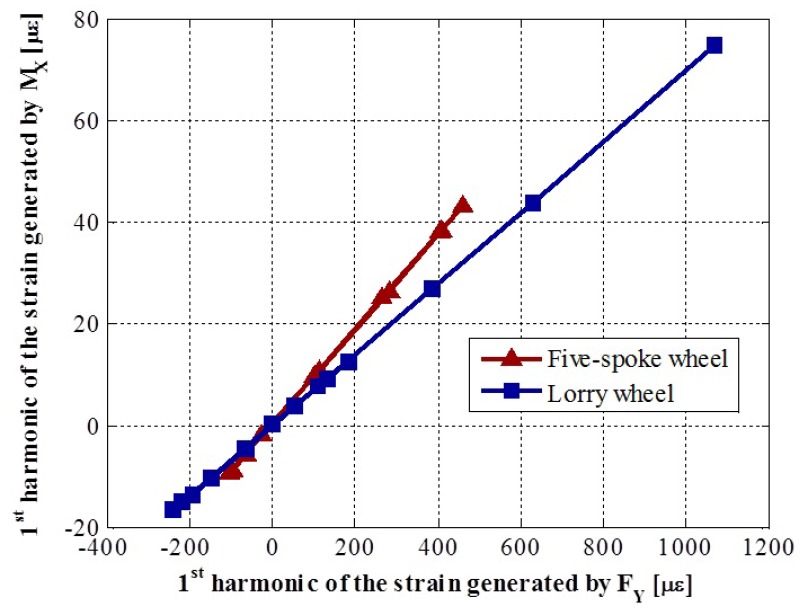
Ratio between the amplitudes of the first harmonic of the strain signals generated by a 150 N m $M_{X}$ moment and a force $F_{Y}$ of 5000 N.

In [12], it was stated that, in order to obtain the symmetric strains, $F_{Z}$, $F_{Y}$ and $M_{X}$, at least three measuring circumferences were needed to compute signal $E_{Si}$. It was further explained that, if the calibration matrix had a conditioning problem, at least one signal dependent on the second harmonic, $E_{Si}^{{}^{\prime }}$, would be necessary. A more detailed study of the finite element models presented in [12] showed that the conditioning problem will always exist, as explained in [13]. Figure 3 shows the proportional relationship between the first harmonic of the influence functions produced by $F_{Y}$ and $M_{X}$, $\varphi_{ij}^{Y}$ and $\psi_{ij}^{X}$, respectively, in both models, lorry and car wheels. This proportionality has been demonstrated for the first harmonic, but it will also apply to higher order harmonics. A physical explanation of this is that when the force $F_{Y}$ is applied at a point different from the real one, for the system to be equivalent, a moment $M_{X}$ needs to be introduced.

Similarly, there will be a proportionality between the strains $F_{X}$ and $M_{Y}$.

### 2.5. Final System of Equations

In order to measure the three forces and three moments on the tyre-road contact area, it is necessary to compute a set of at least three different signals $E_{Si}$ or $E_{Si}^{{}^{\prime }}$ and another set of three different signals $E_{Ai}$ or $E_{Ai}^{{}^{\prime }}$ so that the system of Equation (7) is determined. Therefore, $n_{S}+n_{S}^{{}^{\prime }}\geq 3$ and $n_{A}+n_{A}^{{}^{\prime }}\geq 3$.

Additionally, it is necessary to compute a signal $E_{Ai}^{{}^{\prime }}$ depending on the second harmonic at least in one circumference because, as explained in [12], the $M_{Y}$ strain signal has a second harmonic dominance. Furthermore, as it has been explained in Section 2.4, due to the relationship between the influence functions $\varphi_{ij}^{Y}$ and $\psi_{ij}^{X}$, it is necessary to compute at least a signal $E_{Si}^{{}^{\prime }}$ to ensure that the system (11) is well-conditioned. Therefore, ideally, $n_{S}\geq 2$, $n_{S}^{{}^{\prime }}\geq 1$, $n_{A}\geq 2$ and $n_{A}^{{}^{\prime }}\geq 1$. This is another improvement with respect to the original method presented in [12] because it allows for the number of measuring circumferences to be reduced from three to two, thus increasing the robustness of the system since fewer strain gauges are needed.

## 3. Static Calibration

### 3.1. Semi-Automated Workbench

**Figure 4:**
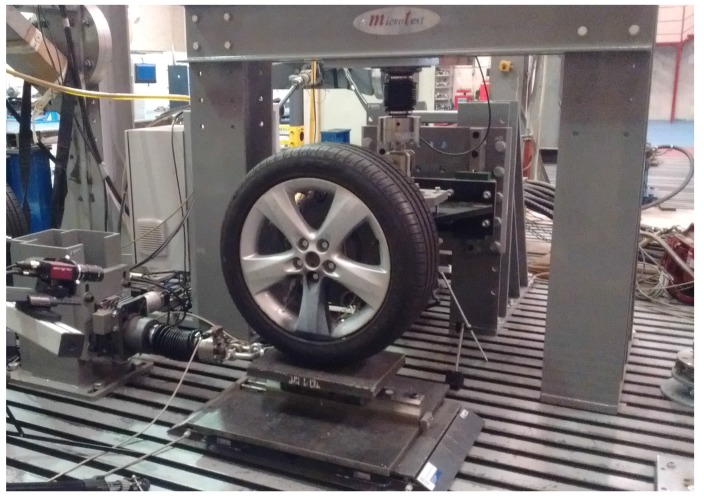
Semi-automatic workbench.

In order to obtain the calibration matrices, $B\left(\alpha \right)$, for measuring the forces and the moments, a set of static experimental tests has to be carried out. For this purpose, a workbench was designed and presented on [12]. In parallel with the work presented in this paper, a new, semi-automated workbench has been designed and built as shown in Figure 4. Nonetheless, for the tests carried out and presented on this paper, the previous workbench has been used because the new one was not still operational.

The semi-automated workbench has three electro-mechanical actuators—100kN in the vertical axis and 50kN in the horizontal ones—and a servomotor that controls the angle turned by the wheel relative to the reference radius, with an accuracy of 0.01 degrees. The main advantages with regards to the older workbench are:

- Substitution of the hydraulic cylinders for electro-mechanical systems that are more accurate, easier to control both in position and force, and therefore with better repeatability.
- Increase in the load capacity of the workbench allowing for testing both car and bus/truck wheels.
- Automation of some of the tasks needed to perform the tests: loading and unloading, rotation of the wheel, braking, etc.

The test procedure is basically the same explained in [12].

The automation of the calibration tests requires the use of both an embedded electronic system and a telemetry system. The previous workbench used wires to connect the strain gauges directly with the data acquisition system but the automatic rotation of the wheel in the new semi-automated workbench does not permit their use.

### 3.2. Embedded Electronics

The embedded electronics are responsible for the acquisition and processing of gauge signals, the computation of forces and the transmission of results to wherever they are going to be used outside of the rim. Due to the specific requirements of the acquisition/computing device, a bespoke design had to be built and tested.

For standard application in a vehicle, rims are built for strength to ensure that they can withstand high load conditions such as bumps or collisions with kerbs. As a result, rims will only suffer very small deformations under normal driving conditions and gauge signals will be weak. For this reason, one of the requirements of the embedded system is that it must be sensitive enough to accurately detect and process such weak strain gauge signals. Furthermore, the quarter bridge gauge configuration, which is often used, has the least possible signal gain. In fact, to detect strain variations of 1με, which is necessary to accurately determine the tyre-road contact forces, the electronics must be capable of measuring voltage differences in the order of μV. To work with such weak signals, a very sensitive set of A/D converters should be used.

In order to estimate tyre-road contact forces, the numerical method presented in 2.1 mathematically combines the frequency characteristics of measured strains, both in their amplitude and phase (vid. Equations (3)–(6)). For this reason, it is crucial that all strain signals be obtained simultaneously for every sample. This means that all the gauges need to be well synchronized. For this reason, electronic solutions based on time multiplexing of channels being sampled were discarded. Such approaches would introduce time lapses between individual channels in the same sample.

It should also be considered that the electronic board will be located in a real vehicle rim and will operate while it is spinning in real road runs, so it will be subjected to challenging and variable environments where exposure to dust, electrical noise, centrifugal forces, huge accelerations and significant temperature changes can be an issue.

**Figure 5:**
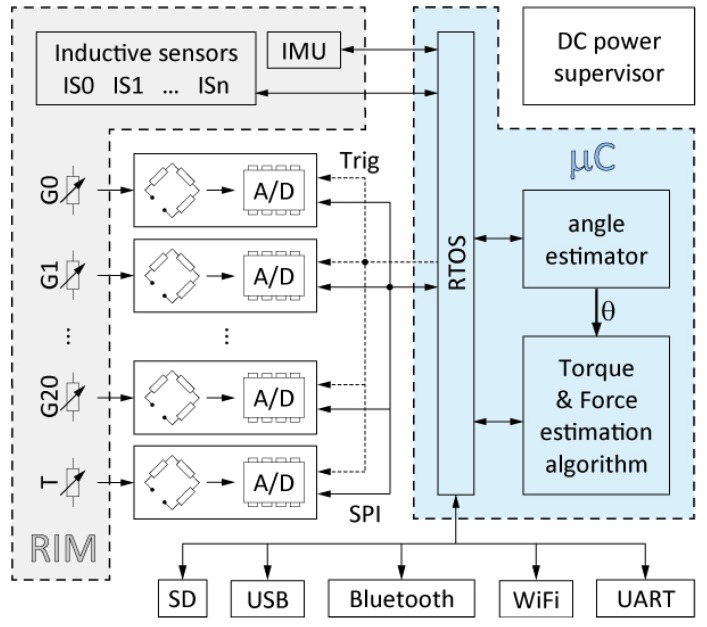
Schematic view of main blocks in the acquisition device.

Finally, a telemetry system based on a Wi-Fi module permits the embedded electronics to communicate with the remote host application. Figure 5 shows a diagram of the different parts composing the electronic board. 

The design criteria required to meet the specifications indicated above can be summarized in the following key points:

- high sensitivity, accuracy, precision and resolution,
- sufficient noise immunity,
- sufficient data acquisition speed,
- synchronous sampling of the whole set of strain gauges located at the measuring points,
- low dependence on temperature variations,
- small size, low weight and low power consumption,
- wired and wireless operating modes and
- sufficient numerical computing power.

**Figure 6:**
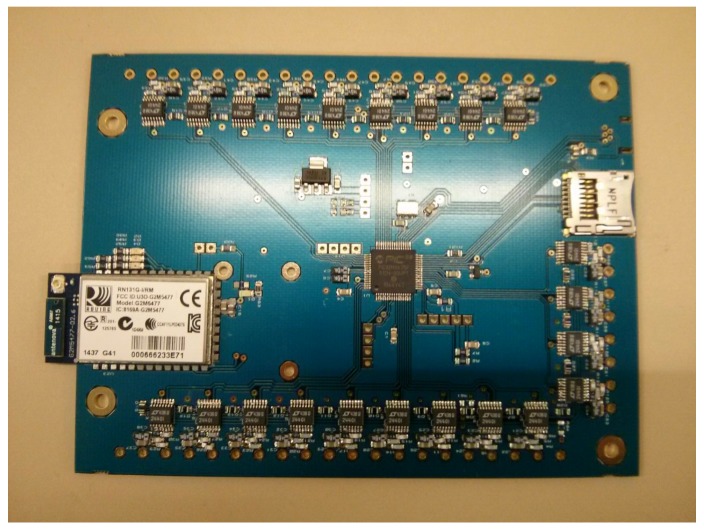
Electronic board prototype designed specifically for the dynamometric wheel.

Figure 6 shows the current electronic board, which is an initial prototype designed following the previous specifications. Currently, another board is under development with a reduced size and some improved characteristics such as a better noise immunity.

### 3.3. Calibration of the Wheel

The wheel used to validate the improvements presented in this paper is a five-spoke Opel Astra© wheel instrumented with five measuring radial lines and three measuring circumferences (see Figure 4). The wheel has been tested applying only a vertical and a lateral force, $F_{Z}$ and $F_{Y}$ in the same way as was explained in [12] (these tests have been performed with the previous workbench presented in [12]). Each test has been replicated to ensure repeatability. Between tests, the wheel has been rotated 9 because this will provide enough points of the influence functions and calibration matrix and is a divisor of 72, which is the angle between spokes. For the intermediate angular positions, interpolation methods are used. This calibration process differs with the usual one of calibrating on a rolling element in order to identify how the values dynamically change as a function of the angular position. However, as the influence functions are the characterization of the strain field of the rim in a specific measurement point, it is something inherent to the rim and, consequently, it is assumed to be independent to dynamic variables as the angular velocity of the rim. Furthermore, as the strain is assumed linear—and in [13] is shown that the assumption holds—the static calibration presented is considered sufficient to be able to estimate the forces and moments.

**Figure 7:**
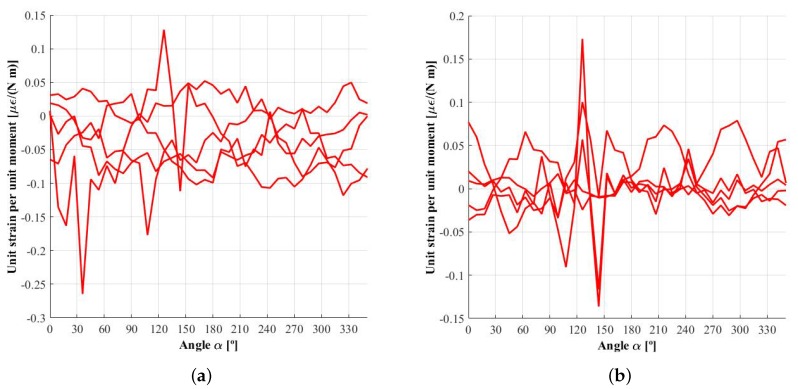
Influence function $\psi_{ij}^{X}$: unit strain produced by a unit moment $M_{X}$. (**a**) inner measuring circumference; (**b**) outer measuring circumference.

**Figure 8:**
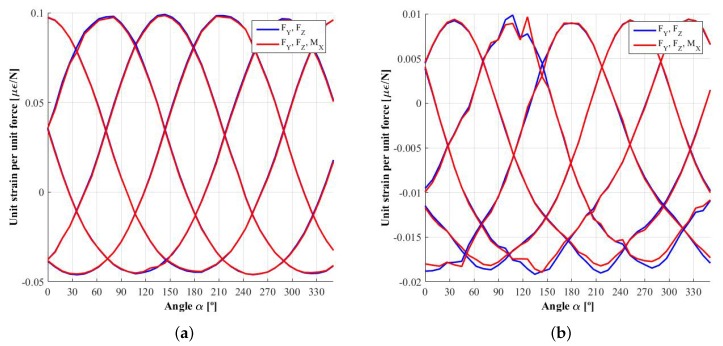
Influence function $\varphi_{ij}^{Y}$: unit strain produced by a unit force $F_{Y}$. (**a**) inner measuring circumference; (**b**) outer measuring circumference.

**Figure 9:**
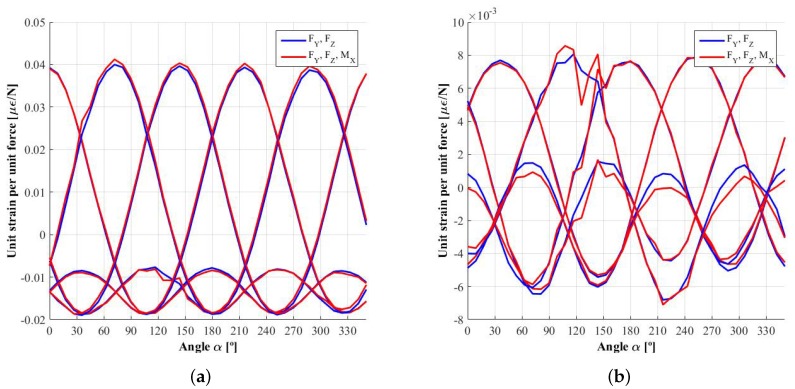
Influence function $\varphi_{ij}^{Z}$: unit strain produced by a unit force $F_{Z}$. (**a**) inner measuring circumference; (**b**) outer measuring circumference.

The tests that measure the effect of $M_{X}$ on the strains and that were explained in [12] have not been performed in this work. Consequently, the effect of the moments has not been taken into account because, without enough data available on its impact, this would only increase the noise on their own influence functions, $\psi_{ij}^{X}$, $\psi_{ij}^{Y}$ and $\psi_{ij}^{Z}$, as can be seen in Figure 7. It would even affect the influence functions associated with the forces, especially in the circumference where the weaker strain signals are measured, as shown in Figure 8 and Figure 9. This is one limitation of the method, although the method remains valid as long as the effect of these moments can be considered negligible, as is the case with smooth manoeuvers where the tyre deformation is not huge.

Figure 9a shows the influence function of force $F_{Z}$, $\varphi_{ij}^{Z}$, measured in the first or innermost circumference. It can be seen that, due to factors such as the lack of precision in gauge installation and/or asymmetries introduced during the rim manufacturing process, the signals obtained in each spoke are not completely equivalent, which was one of the symmetry requirements of the method. As a result, since the harmonic composition is not exactly the same, the ripple in the demodulated signals $E_{Si}$, $E_{Ai}$, $E_{Si}^{{}^{\prime }}$ and $E_{Ai}^{{}^{\prime }}$ will be non-negligible, especially in the second harmonic dependent signals $E_{Si}^{{}^{\prime }}$ and $E_{Ai}^{{}^{\prime }}$.

In order to validate the improvements presented in [13], several configurations of the dynamometric wheel have been assessed:

- Fifteen strain gauges distributed amongst five measuring radial lines and three measuring circumferences.
- Minimum set of strain gauges: two measuring circumferences and five radial lines. Furthermore, in order to study the influence of the stiffness of the rim with this method, two of the three possible circumference combinations have been studied: the best option was to use the circumferences closest to the centre of the wheel, where the strain is greater; and the worst one was to use the outer two circumferences.

Furthermore, a calibration method that assumes the ripple to be negligible following [12] has also been used to study the resulting errors.

## 4. Experimental Results

### 4.1. Relationship between the Amplitudes of the Strain Signals Generated by $M_{X}$ and $F_{Y}$

**Figure 10:**
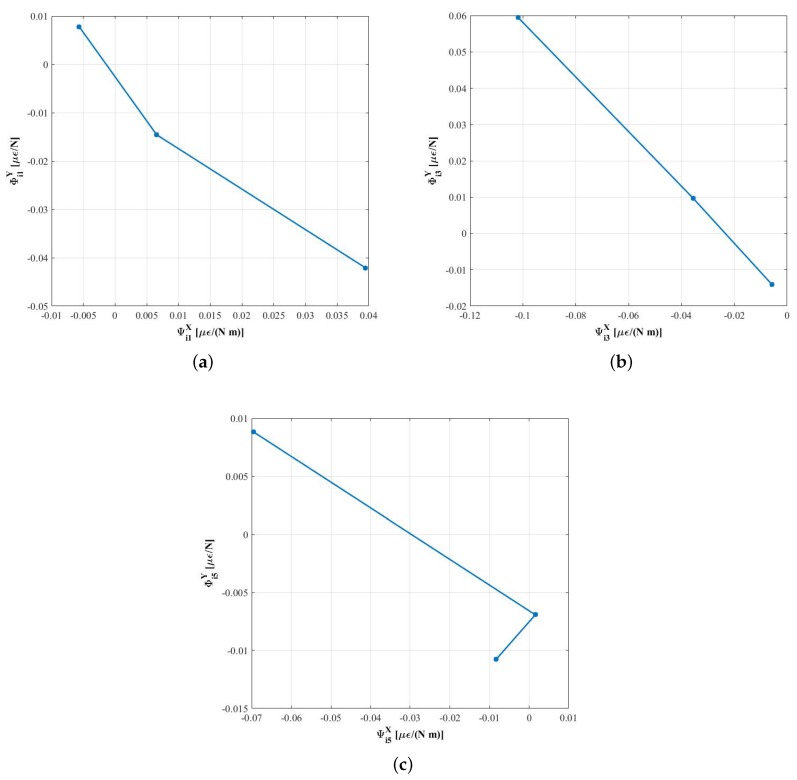
Relationship between the influence functions $\psi_{ij}^{X}$ and $\varphi_{ij}^{Y}$. (**a**) measuring radial line 1; (**b**) measuring radial line 3; (**c**) measuring radial line 5.

The relationship between the influence functions explained in 2.4 and studied with a finite element model was tested using experimental data obtained in the set of calibration tests. The results for three of the five measuring lines are shown in Figure 10. Each of the points in the figure correspond to one of the measuring circumferences. The linear relationship between the influence functions obtained in each circumference is easily seen in Figure 10b. However, the linear relationship is not so obvious in Figure 10a and even nonexistent in Figure 10c. This is due to high noise in the influence functions corresponding to the moment $M_{X}$, $\psi_{ij}^{X}$, which, in turn, results from not having made the moment’s tests for a good characterization of these signals, as was explained in 3.3 and shown in Figure 7.

### 4.2. Demodulated Signals and Influence of the Higher Order Harmonics

**Figure 11:**
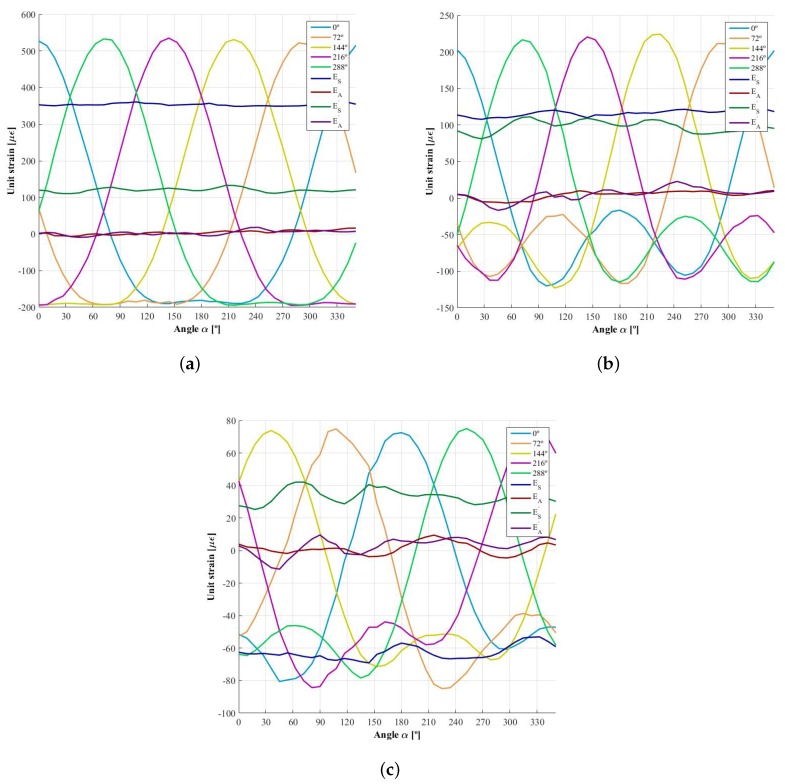
Strain signals generated when a combination of forces $F_{Z}=6000$ N and $F_{Y}=3000$ N acts at he contact patch and the demodulated signals corresponding to the first and second harmonic, $E_{Si}$, $E_{Ai}$, $E_{Si}^{{}^{\prime }}$ and $E_{Ai}^{{}^{\prime }}$. (**a**) measurement circumference 1 (inner); (**b**) measurement circumference 2; (**c**) measurement circumference 3 (outer).

Once the influence functions, $\varphi_{ij}^{Y}$ and $\varphi_{ij}^{Z}$, have been obtained, it is possible to calculate *virtual* strain signals known *virtual* forces, $F_{Z}$ and $F_{Y}$, and using (1). Knowing the angle rotated by the wheel and combining the strain signals as per (3)–(6), the demodulated signal can be determined. In Figure 11, the strain signals obtained for a force $F_{Z}$ of 6000 N and $F_{Y}$ of 3000 N are shown as well as the demodulated signals $E_{Si}$, $E_{Ai}$, $E_{Si}^{{}^{\prime }}$ and $E_{Ai}^{{}^{\prime }}$. Due to the fact that the forces simulated are symmetrical, $E_{Ai}$ and $E_{Ai}^{{}^{\prime }}$ oscillate around the null value. However, the most important observation is that the fifth harmonic is not negligible, especially on the second harmonic dependent signals, $E_{Si}^{{}^{\prime }}$ and $E_{Ai}^{{}^{\prime }}$. Table 1 shows the average values of the demodulated signals with their percentage variation. The huge variation in the *antisymmetrical* signals is due to oscillation around a very small value, near zero. Furthermore, the percentage increases with the number of measuring circumferences, corresponding to a weaker sensitivity in the influence functions. In addition, the variance is higher in the signals corresponding to the second harmonic.

**Figure 12:**
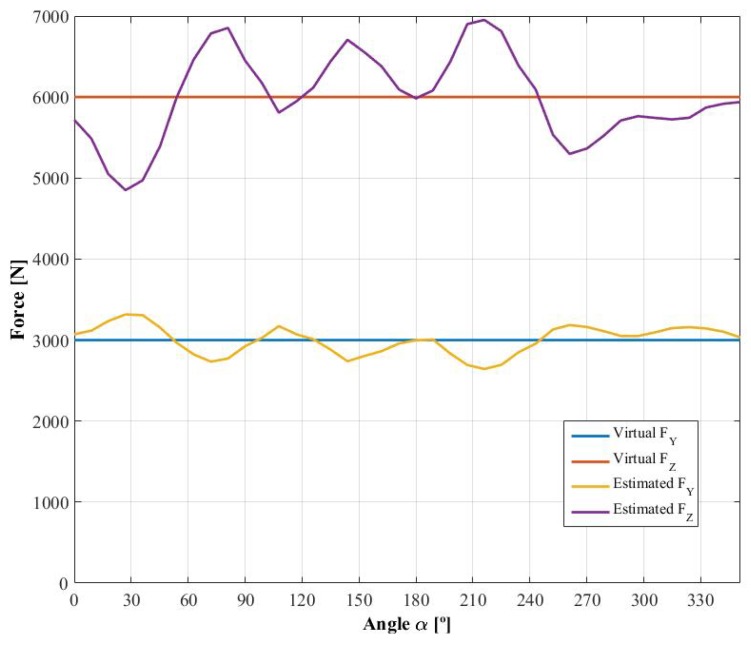
*Virtual* and estimated forces.

Finally, in Figure 12, the estimated forces obtained using the constant calibration matrix (analogous to the one presented in [12]) are shown as well as the *virtual* force used to generate the strain signals. The constant calibration matrix for the fifteen measurement points is:

(12) $$\left[\begin{array}{ccc} 0 & 0.0729 & 0.0225\\ 0 & 6.8109\times 10^{-4} & 3.4377\times 10^{-4}\\ 0 & 0.0216 & 0.0086\\ 0 & 8.5200\times 10^{-4} & 2.9181\times 10^{-4}\\ 0 & -0.0129 & -0.0040\\ 0 & 2.1746\times 10^{-4} & 1.0569\times 10^{-4}\\ 0 & 0.0086 & 0.0157\\ 0 & 1.7693\times 10^{-4} & 4.8916\times 10^{-4}\\ 0 & 0.0067 & 0.0129\\ 0 & 3.7407\times 10^{-4} & 6.9114\times 10^{-4}\\ 0 & 0.0024 & 0.0044\\ 0 & 1.5270\times 10^{-4} & 3.8416\times 10^{-4} \end{array}\right]\left\{\begin{array}{c} M_{X}\left(t\right)\\ F_{Y}\left(t\right)\\ F_{Z}\left(t\right) \end{array}\right\}=\left\{\begin{array}{c} E_{S1}\\ E_{A1}\\ E_{S2}\\ E_{A2}\\ E_{S3}\\ E_{A3}\\ E_{S1}^{{}^{\prime }}\\ E_{A1}^{{}^{\prime }}\\ E_{S2}^{{}^{\prime }}\\ E_{A2}^{{}^{\prime }}\\ E_{S3}^{{}^{\prime }}\\ E_{A3}^{{}^{\prime }} \end{array}\right\},$$

where the column corresponding to the moment $M_{X}$ is null because, as explained in 3.3, the influence of the moments has not been considered.

### 4.3. Errors While Estimating Forces

**Figure 13:**
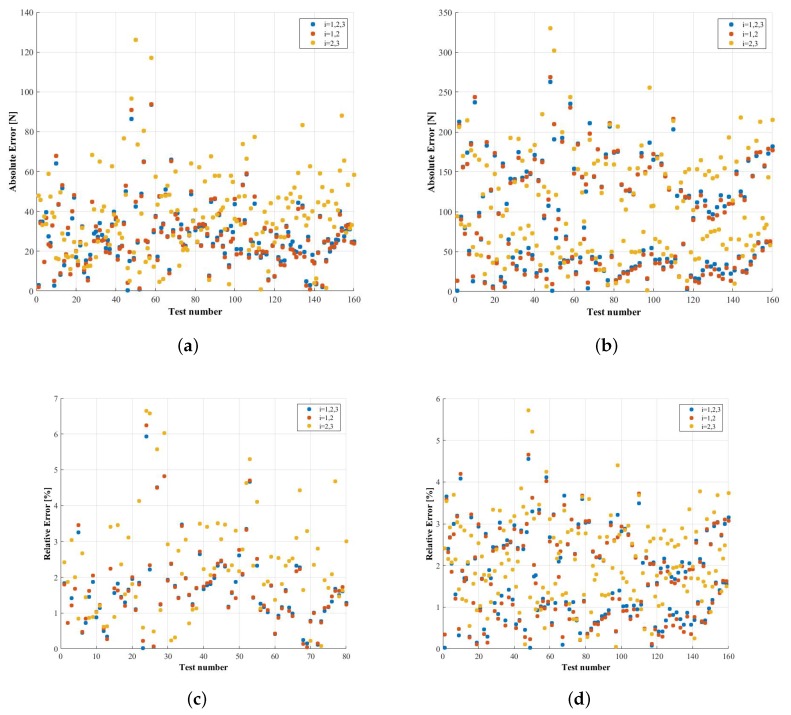
Comparison between the absolute and relative errors obtained when using matrices depending on the angular position using different measurement circumferences (new method proposed). (**a**) absolute error in the measurement of $F_{Y}$; (**b**) absolute error in the measurement of $F_{Z}$; (**c**) relative error in the measurement of $F_{Y}$; (**d**) relative error in the measurement of $F_{Z}$.

Some of the test results from the calibration workbench have not been used to obtain the influence functions and the calibration matrices. These results have instead been used to validate and estimate the expected errors by comparing the measurement of the load cell integrated in the actuators of the workbench with the force estimated according with the method presented in this research. A comparison between the different configurations explained in 3.3 has been made to determine the best way of instrumenting the tire. Figure 13 shows the absolute and relative errors and Table 2 and Table 3 show the maximum and mean values of the relative errors obtained using the three options studied when estimating $F_{Y}$ and $F_{Z}$, respectively.

It can be seen that the option based on two measuring circumferences has approximately the same mean error as that associated with using three circumferences, around 1.7% when estimating $F_{Y}$ and less than 1.8% in the case of $F_{Z}$. Therefore, it is feasible to reduce the number of measuring circumferences without any related relevant increase in the estimation error. Between choosing the circumferences with a higher strain signal or the ones with a weaker one, there is no statistically significant difference, although the errors are somewhat higher in the latter case (2.4% error instead of 1.7% when estimating $F_{Y}$ and 2.1% error instead of 1.7% in the case of $F_{Y}$). The maximum errors are less than 7% and are caused mostly by the noise in the signals. Currently, efforts are being made to improve the design of the analog front-end in order to ensure a higher resolution and noise immunity of the strain signals with a better signal acquisition and conditioning unit. As it was previously stated, the stiffness of the rims is really high and consequently the gauge signals are weak.

**Figure 14:**
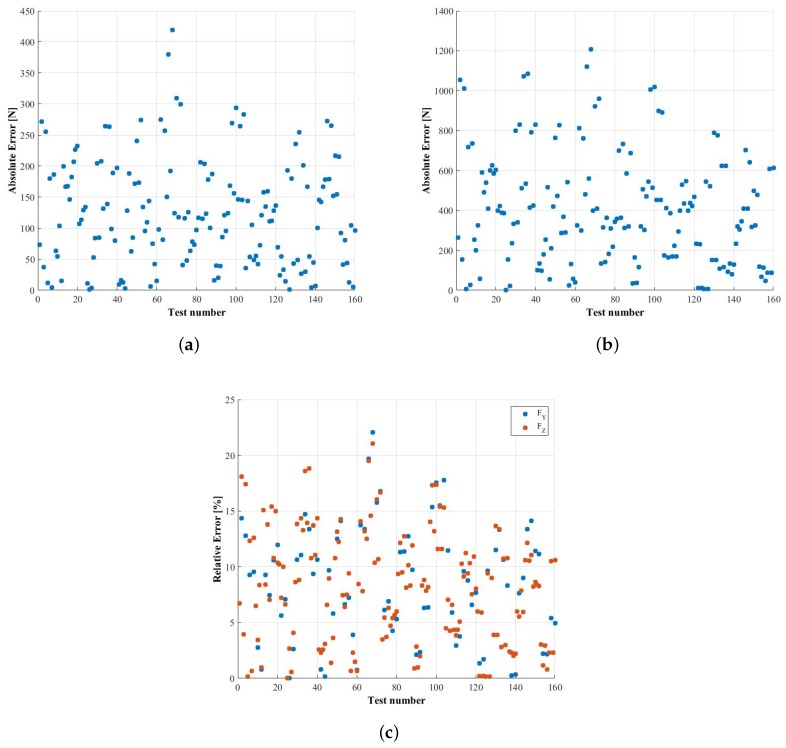
Absolute and relative errors obtained when using the superseded constant matrix method. (**a**) absolute error in the measurement of $F_{Y}$; (**b**) absolute error in the measurement of $F_{Z}$; (**c**) relative error in the measurement of $F_{Y}$ and $F_{Z}$.

On the other hand, in Figure 14, the absolute and relative errors calculated for the case where the constant calibration matrix is used are presented. The maximum relative error obtained when estimating $F_{Y}$ is 22.065%, very similar to the 21.0441% obtained when estimating $F_{Z}$. The mean relative errors of the estimation of $F_{Y}$ and $F_{Z}$ are 8.6563% and 8.1652%, respectively. Therefore, the constant calibration matrix presented in [12] has some limitations when applied to the case of wheels with fewer spokes or at least when the higher order harmonics are not negligible and consequently is superseded by the new method proposed in the present paper.

## 5. Conclusions

In the present paper, the effect of reducing the number of radial lines in the method presented in [12] has been shown: the ripple due to the higher harmonics that cannot be canceled is non-negligible and therefore a calibration depending on the angle is proposed. This method also solves other symmetry problems such as the lack of precision in the installation of the strain gauges and minor manufacturing deviations in the rim. In order to provide sufficient data to describe the influence functions and calibration matrices, a set of static tests are performed every 9, and the intermediate angular positions are interpolated.

It has also been proved that it is not necessary to use three measuring circumferences because due to the linear relationship between the influence functions corresponding to $F_{Y}$ and $M_{X}$, it is not possible to estimate the symmetrical forces using only signals that depend on the first harmonic as was proposed in [12]. Furthermore, it has been proved that there is not a significant difference between using three or two measuring circumferences. Consequently, a reduced number of measuring points can be implemented reducing the cost and increasing the robustness of the system.

Finally, the new semi-automated calibration workbench has been presented along with the embedded electronic system needed to acquire and process the strain signals and transmit them wirelessly.

## Figures and Tables

**Table 1:** Values of the demodulated signals.

	i=1	i=2	i=3
$E_{S}$	353.79 ± 2.28%	116.41 ± 7.54%	−62.70 ± 15.41%
$E_{A}$	4.11 ± 297.39%	4.31 ± 253.22%	1.29 ± 644.85%
$E_{S}^{{}^{\prime }}$	120.10 ± 10.83%	97.83 ± 17.04%	33.42 ± 25.91%
$E_{A}^{{}^{\prime }}$	3.47 ± 434.61%	5.27 ± 415.75%	2.76 ± 511.45%

**Table 2:** Mean and maximum value of the relative error when estimating $F_{Y}$.

	i=1,2,3	i=1,2	i=2,3
$\bar{e_{\alpha }}$	1.6768%	1.6880%	2.3793%
$\;maxe_{\alpha }$	5.9297%	6.2446%	6.6426%

**Table 3:** Mean and maximum value of the relative error when estimating $F_{Z}$.

	i=1,2,3	i=1,2	i=2,3
$\bar{e_{\alpha }}$	1.7651%	1.7161%	2.0885%
$\;maxe_{\alpha }$	4.5502%	4.6518%	5.7134%
